# Metabonomic Analysis Provides New Insights into the Response of Zhikong Scallop (*Chlamys farreri*) to Heat Stress by Improving Energy Metabolism and Antioxidant Capacity

**DOI:** 10.3390/antiox11061084

**Published:** 2022-05-30

**Authors:** Xixi Dong, Zujing Yang, Zhi Liu, Xuefeng Wang, Haitao Yu, Cheng Peng, Xiujiang Hou, Wei Lu, Qiang Xing, Jingjie Hu, Xiaoting Huang, Zhenmin Bao

**Affiliations:** 1 MOE Key Laboratory of Marine Genetics and Breeding, College of Marine Life Sciences, Ocean University of China, Qingdao 266003, China; 18334789706@163.com (X.D.); yzj@ouc.edu.cn (Z.Y.); liuzhiouc@163.com (Z.L.); wangxf13210173557@163.com (X.W.); haitao0532@foxmail.com (H.Y.); pengcmiles@163.com (C.P.); houxiujiang@stu.ouc.edu.cn (X.H.); lw1981@ouc.edu.cn (W.L.); qiangxing@ouc.edu.cn (Q.X.); hujingjie@ouc.edu.cn (J.H.); zmbao@ouc.edu.cn (Z.B.); 2 Laboratory for Marine Fisheries Science and Food Production Processes, Qingdao 266237, China; 3 Laboratory of Tropical Marine Germplasm Resources and Breeding Engineering, Sanya Oceanographic Institution of the Ocean University of China (SOI-OUC), Sanya 572000, China

**Keywords:** *Chlamys farreri*, heat stress, energy metabolism, antioxidant capacity

## Abstract

Temperature is an important factor affecting the growth, development and survival of marine organisms. A short episode of high temperature has been proven to be a severe threat to sustainable shellfish culture. Zhikong scallop (*Chlamys farreri*), a shellfish with broad economic and biological value in North China, has frequently experienced heat stress in summer in recent years. To understand the effects of heat stress on shellfish, the metabolism of *C. farreri* was analyzed after exposure to 27 °C for either 6 h or 30 d. After 6 h of heat stress exposure, a total of 326 and 264 significantly different metabolites (SDMs) were identified in gill and mantle tissues, respectively. After 30 d of heat stress exposure, a total of 381 and 341 SDMs were found in the gill and mantle tissues, respectively. These SDMs were mainly related to the metabolism of amino acids, carbohydrates, lipids and nucleotides. A decline in pyruvic acid, and an increase in citric acid and fumaric acid in the gills and mantle of *C. farreri* indicated an alteration in energy metabolism, which may be attributed to increased ATP production in order to overcome the heat stress. Among the SDMs, 33 metabolites, including pyruvic acid, glycine and citric acid, were selected as potential biomarkers for heat stress response in *C. farreri*. In addition, a decline in glutamine and β-Alanine levels indicated oxidative stress in *C. farreri* exposed to heat, as well as an increase in the total antioxidant capacity (T-AOC). Our findings suggested *C. farreri* have the potential to adapt to heat stress by regulating energy metabolism and antioxidant capacity.

## 1. Introduction

In recent years, extreme weather conditions, especially high temperatures, threatening the survival of nearly all marine organisms have occurred frequently around the world [1,2]. Several researchers have suggested that heat stress adversely affects all aquatic organisms through various mechanisms, including the disturbance of energy metabolism, mitochondrial disorders and oxidative stress [3,4,5,6]. Further studies to elucidate the adaptive potential of living creatures and explain their performance in current environments will obviously aid our knowledge of the response of organisms to heat stress in the future.

Metabolomics aims to investigate the changes to metabolites in biological systems (cells, tissues, etc.) after stimulation or disturbance. Recently, metabolomics has been widely applied, especially in studies investigating the responses of organisms to external stimuli or pathogenic challenges [7,8,9]. For example, comparative metabolic analysis has demonstrated that altered lipid profiles, increases in toxic substances and reduced genetic material synthesis may contribute to the high mortality of *Strongylocentrotus intermedius* under seawater acidification conditions [10]. Li et al. investigated summer mortality of *Perna canaliculus* and found disruption of the tricarboxylic acid (TCA) cycle and fatty acid metabolism in unhealthy mussels through metabolomics-based analyses [5]. It was found through LC-MS metabolomics studies that in yellow drum (*Nibea albiflora*), glutathione metabolism and its related metabolites (glutamate and GSSG) could be potential biomarkers of cold and starvation stresses [11]. In addition, there have been many studies on the effects of high temperature on marine organisms and their response mechanisms from the perspective of metabonomics. For instance, Zhang et al. researched the effects of temperature on the growth and development of European seabass (*Dicentrarchus labrax*) using metabolomics, and found that the growth rate was highest in the heat-treated group; they noted changes of pathway related to growth and development under heat stress [12]. Metabolome analysis has revealed the effects of thermal stress on lipid metabolism in juvenile turbot (*Scophthalmus maximus*), which was found to regulate lipid deposition and homogenization by altering the concentration of metabolites [13], and that these creatures had the potential to adapt to changes in temperature.

When exposed to natural disturbances (e.g., temperature rise), bivalves may produce oxygen radicals, which leads to changes in antioxidant enzymes such as superoxide dismutase (SOD), catalase (CAT), glutathione S-transferases activities (GST) and others [14,15]. For example, in Mizuhopecten yessoensis and Haliotis discus hannai, the activities of SOD and CAT first significantly increased under heat stress, and then returned to the level of the control group with the extension of stress time [16]. SOD and CAT have been select-ed as biomarkers of the health status of bivalves [17,18]. These studies indicated that bivalves have a certain ability to recover in a short period of time after suffering from oxidative damage, and that these creatures had the potential to adapt to changes in temperature.

Zhikong scallop, *C. farreri* (Jones, 1904, also known as Chinese scallop), is naturally distributed along the coasts of Northern China, Korea, Japan and Eastern Russia. They usually live a semisessile lifestyle, attaching to rocks and other hard surfaces using byssal threads [19]. *C. farreri* is one of the most important commercially farmed shellfish; in north China in 2019, the annual yield of scallops exceeded 1.83 million tons, providing a major contribution to national mariculture production [20,21]. Previous research has mainly fo-cused on the effects of high temperature on *C. farreri* at a molecular level [22,23]. The mac-roscopic description, from a metabolomics perspective, is still absent, but this may pro-vide powerful information to understand the response mechanism of *C. farreri* under heat stress.

In this study, we utilized untargeted mass spectrometry coupled with liquid chro-matography (LC-MS) to explore metabolic changes in *C. farreri* under heat stress. In addi-tion, T-AOC, SOD, CAT, GST, malondialdehyde (MDA) and ATP concentrations were in-vestigated to reveal the antioxidant capacity and energy levels of *C. farreri* under heat stress. The results will assist in deciphering the mechanisms for the regulation of heat stress in *C. farreri*.

## 2. Materials and Methods

### 2.1. Sample Collection and Preparation

In October 2018, healthy Zhikong scallops (shell height: 54.60 ± 3.86 mm) were collected from Qingdao (Shandong Province, China) and transported alive to the laboratory. The scallops were acclimatized for a week in a recirculating culture system (temperature 19 °C, pH 8.0 and salinity 30.0‰). Each day, the seawater was replaced and the scallops were fed with fresh diatoms (*Nitzschia closterium*, 10^6^ cells/scallop). After acclimation, 90 healthy scallops were divided into three 50 L tanks and exposed to 27 °C seawater, which corresponded to the maximum temperature in the distribution area of *C. farreri*. In the 27 °C group, nine scallops were randomly sampled at 6 h and 30 d, which represented the short- and long-period heat stress experiments, respectively. The remaining scallops were cultured in seawater at 19 °C and sampled as controls. The mantle and gill tissue from each scallop, which was thought to be sensitive to environmental changes, was dissected and frozen in nitrogen for further experiments.

### 2.2. Metabolite Extraction and Profiling

Tissue samples (100 mg) were individually ground with liquid nitrogen, and the homogenate was resuspended with prechilled 80% methanol and 0.1% formic acid using a vortex mixer. The samples were incubated on ice for 5 min, and then centrifuged at 15,000 rpm at 4 °C for 5 min. The supernatant was diluted to final concentration with LC-MS grade water containing 53% methanol. The samples were subsequently transferred to a fresh Eppendorf tube and then they were centrifuged at 15,000× *g* at 4 °C for 10 min. Finally, the supernatant was injected into the LC-MS/MS system for analysis.

The raw data files generated by UHPLC-MS/MS were processed using Compound Discoverer 3.1 software (CD 3.1, Thermo Fisher, Waltham, MA, USA) to perform peak alignment, peak picking and quantitation for each metabolite. Peaks were matched with the mzCloud (https://www.mzcloud.org/, accessed on 2 December 2020), mzVault and MassList databases to obtain accurate qualitative and relative quantitative results for the metabolites. These metabolites were annotated using the Lipidmaps (http://www.lipidmaps.org/, accessed on 2 December 2020) and KEGG databases (https://www.genome.jp/kegg/, accessed on 2 December 2020).

### 2.3. Biochemical Analysis

In this study, T-AOC, SOD, CAT and GST activities, MDA concentration and ATP content were measured with test kits (Nanjing Jiancheng Bioengineering Institute, Nanjing, China). The T-AOC was estimated using the FRAP method [24]. The SOD activity was measured using the WBT-1 method [25]. The CAT activity was determined on the basis of the ammonium molybdate method [26]. The GST activity was obtained according to the method of Habig et al. [27]. The quantitative measurement of MDA was based on the reaction of MDA and thiobarbituric acid (TBA). The reaction product, MDA-TBA2, was measured at 532 nm [28]. The ATP content was determined according to the phosphomolybdic acid colorimetry method [29].

### 2.4. Statistical Analysis

Principle component analysis (PCA) using an unsupervised method provided an overview of the metabolic data, general clustering, trends and visualization of outliers. We applied univariate analysis (*t*-test) to calculate the statistical significance (*p*-value). The metabolites having the first principal component of the variable importance projection (VIP) >1 obtained from partial least squares discrimination analysis (PLS-DA), *p*-value < 0.05 and fold change (FC) > 1.2 or FC < 0.833 were considered to be significantly different metabolites (SDMs). The quantitative relationships of the SDMs were analyzed by TBtools [30]. In order to represent the relative content and relationships of some pivotal SDMs in the experimental groups, cluster analysis was conducted using OmicStudio (https://www.omicstudio.cn/tool/4, accessed on 15 December 2021).

Statistical analyses were conducted using one-way analysis of variance followed by post hoc comparison of a means-based Tukey’s honestly significant difference (HSD) test, using IBM SPSS Statistics 20 software (IBMCorp, Armonk, NY, USA); the significance level was set at *p* < 0.05.

## 3. Results

### 3.1. Metabolic Profile of C. farreri under Heat Stress

**Figure 1:**
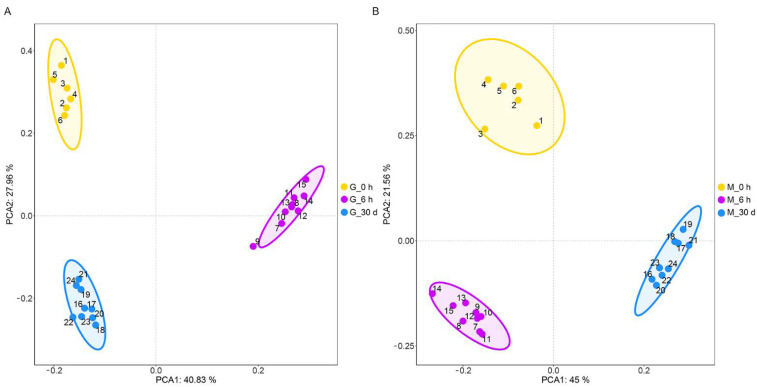
Principal component analysis (PCA) in gill (**A**) and mantle (**B**) of *C. farreri* under heat stress for 6 h and 30 d. Yellow dots: control (heat stress for 0 h); purple dots: heat stress for 6 h; blue dots: heat stress for 30 d.

Differences among groups were easily detected using unsupervised PCA of mantle and gill tissues from *C. farreri* subjected to heat stress (Figure 1). All samples in the score plots were within the 95% confidence ellipses of Hotelling’s T-squared distribution, and no significant outliers were found in the PCA scores plots of each sample, indicating our data had high homogeneity. Furthermore, clear separation and discrimination were found between the pairwise groups, indicating the metabolites found in the gill and mantle tissues of *C. farreri* were significantly different among the different stress times.

**Figure 2:**
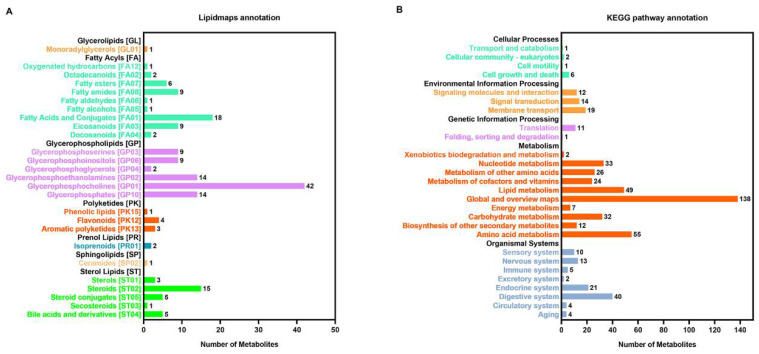
Annotation of metabolites of *C. farreri* under heat stress by Lipidmaps (**A**) and KEGG pathways (**B**). The *X*-axis represents the number of metabolites. The *Y*-axis represents the annotated Lipidmaps or KEGG terms.

A total of 1048 metabolites were obtained from each sample. Lipidmaps annotation (Figure 2A) showed that the top three metabolites were glycerophosphocholines, fatty acids and their conjugates, and steroids. The functions of the metabolites were mainly related to the metabolism of amino acids, carbohydrates, lipids and nucleotides, as annotated by KEGG (Figure 2B).

### 3.2. Heat Stress Response—Significantly Different Metabolites and Metabolic Pathways

**Figure 3:**
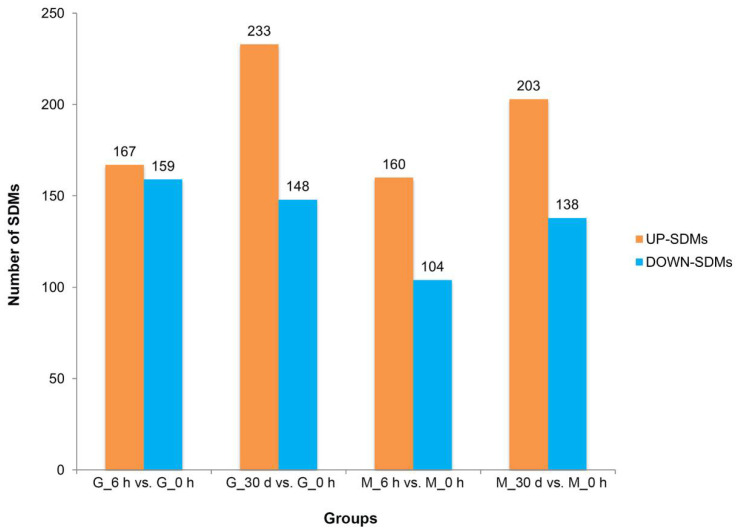
Significantly different metabolites (SDMs) in gill (G) and mantle (M) tissues of *C. farreri* subjected to heat stress for 6 h and 30 d are shown in the column chart. The orange columns represent the number of up-regulated SDMs, and the blue columns represent the number of down-regulated SDMs.

**Figure 4:**
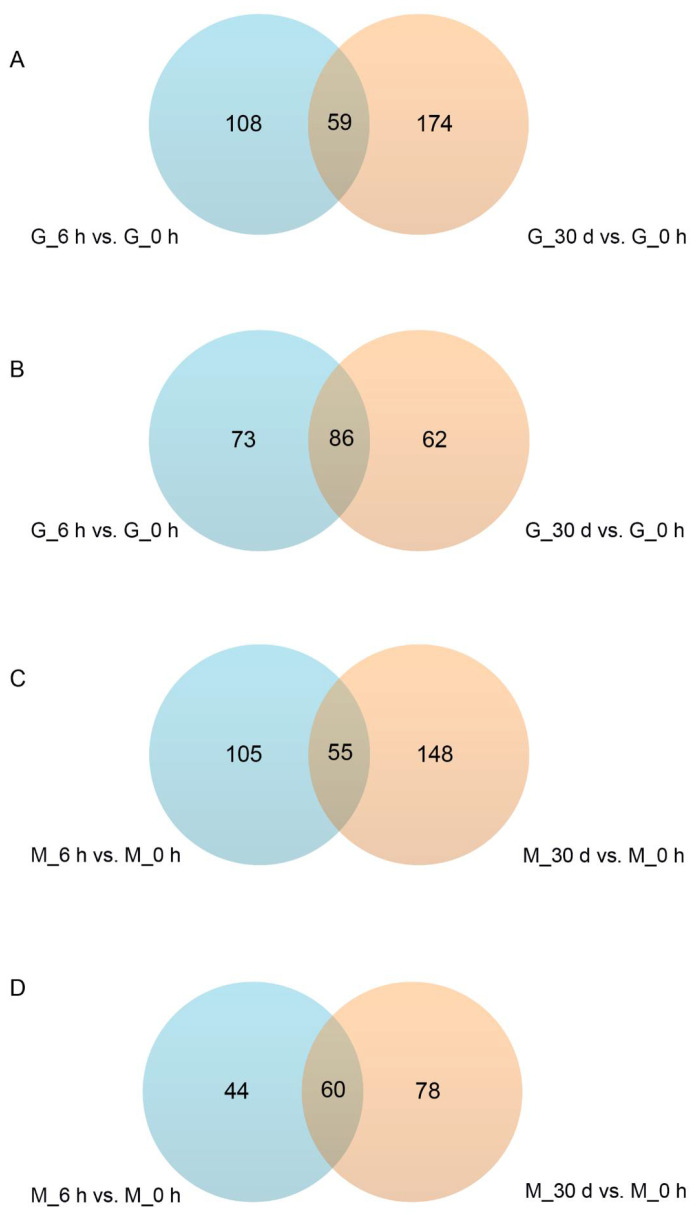
The Venn diagram shows that the significantly different metabolites (SDMs) overlap in each comparison. (**A**): up-regulated SDMs overlap in G_6 h vs. G_0 h and G_30 d vs. G_0 h. (**B**): down-regulated SDMs overlap in G_6 h vs. G_0 h and G_30 d vs. G_0 h. (**C**): up-regulated SDMs overlap in M_6 h vs. M_0 h and M_30 d vs. M_0 h. (**D**): down-regulated SDMs overlap in M_6 h vs. M_0 h and M_30 d vs. M_0 h.

Under heat stress for 6 h, a total of 326 and 264 SDMs were identified in the gill and mantle tissues, respectively. Among them, 167 SDMs were upregulated and 159 were downregulated in gill tissue, and 160 SDMs were upregulated and 104 were downregulated in mantle tissue. Under heat stress for 30 days, *C. farreri* showed alterations in 381 SDMs (increases in 233 and decreases in 148) in gill tissue, and in 341 SDMs (increases in 203 and decreases in 138) in mantle tissues (Figure 3). In addition, 59 SDMs were up-regulated and 86 SDMs were down-regulated in gill tissue during heat stress, while 55 SDMs were up-regulated and 60 SDMs were down-regulated in mantle tissues during heat stress (Figure 4).

Furthermore, these SDMs were classified into their respective biochemical pathways, as described by the KEGG database. The pathway enrichment results are shown in Table 1. The analysis suggested that the metabolites responding to high temperature mainly participate in 10 target pathways (*p* < 0.05), including the TCA cycle, pentose and glucoronate interconversions, aminoacyl-tRNA biosynthesis, sulfur metabolism, primary bile acid biosynthesis and amino acid metabolism.

**Figure 5:**
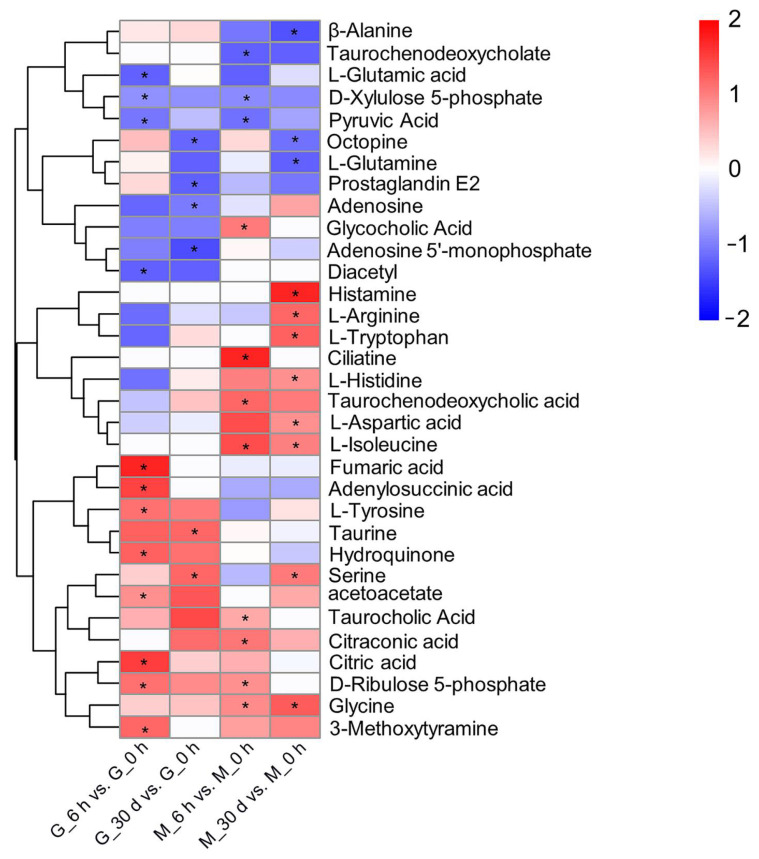
Heatmap plot of significantly different metabolites (SDMs) annotated by KEGG enrichment pathway analysis in gill (G) and mantle (M) tissues of *C. farreri* subjected to heat stress for 6 h and 30 d. Significance * *p* < 0.05.

In addition, we performed cluster analysis on the SDMs in relation to the pathways mentioned above, and the results are shown in Figure 5. The metabolites, such as citric acid and fumaric acid, which are involved in the TCA cycle increased significantly in gill tissue under heat stress for 6 h, while the level of pyruvic acid, which participates in the TCA cycle, and interconversions between pentose and glucoronate decreased sharply in both mantle and gill tissues under heat stress for 6 h. The concentrations of free amino acids involved in aminoacyl-tRNA biosynthesis and amino acid metabolism, except for L-glutamine and β-alanine, were elevated following heat stress. Serine and taurine are related to sulfur metabolism. Serine increased significantly in gill and mantle tissues under heat stress for 30 d, while taurine rose significantly only in gill tissues under heat stress for 30 d. The primary bile acid biosynthesis pathway involves glycine and glycocholic acid, which both increased significantly in mantle tissues under heat stress.

### 3.3. Effects of Heat on Biochemical Indexes in C. farreri

**Figure 6:**
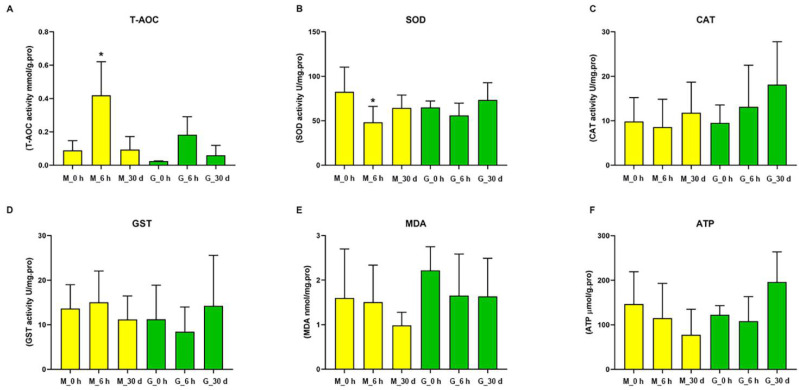
Antioxidant activity and the content of MDA and ATP in mantle and gill tissues of *C. farreri* under heat stress for 6 h and 30 d. (**A**) total antioxidant capacity (T-AOC); (**B**) superoxide dismutase (SOD); (**C**) catalase (CAT); (**D**) glutathione S-transferases (GST); (**E**) malondialdehyde (MDA); (**F**) ATP. * denotes significant differences from the control group.

The biochemical indexes, T-AOC, SOD, CAT and GST activities, MDA concentration and ATP content, were used to evaluate the effect of heat stress on *C. farreri*. Figure 6 shows that T-AOC increased significantly in mantle tissues after heat stress for 6 h. No significant changes were observed in the other groups. In contrast, the activity of SOD in mantle tissue was obviously decreased following heat stress for 6 h. The activity of CAT increased, but the level of MDA decreased in both mantle and gill tissues with the extension of stress time. The ATP concentration and GST activity decreased in mantle tissue, but increased in gill tissue, with the extension of stress time. The concentrations of MDA and ATP, and the CAT and GST activities did not change significantly during the heat stress period.

## 4. Discussion

In the present study, we have reported for the first time, metabolic alterations in mantle and gill tissue from *C. farreri* subjected to heat stress, determined using an LC-MS/MS-based metabolomics approach, in order to provide a theoretical basis for the adjustment mechanisms of *C. farreri* under heat stress. Metabolite profiles of the mantle and gill tissues of *C. farreri* showed a large number of SDMs that suggest a significant physiological response caused by the heat stress. The number of up-regulated SDMs was greater than the number of down-regulated SDMs in all treatment groups. This phenomenon indicates, to a certain extent, that metabolic consumption in *C. farreri* increases under heat stress; which is consistent with the studies which reported metabolite changes that showed 21 SDMs were up-regulated and 15 SDMs were down-regulated in *Sebastes schlegelii* stimulated by acute high temperature (27 °C), and which demonstrated that 57 SDMs were elevated and 30 SDMs were decreased in *S. intermedius* in acidified seawater treatment groups [10,31].

### 4.1. Energy Metabolism

Short-term heat stress affected carbohydrate and amino acid metabolism in both the mantle and gill tissues of *C. farreri*. Pyruvic acid, as the final product of glycolysis, can be converted into acetyl-CoA under aerobic conditions and enter the tricarboxylic acid (TCA) cycle. The TCA cycle is a crucial pathway for cellular energy metabolism. In our study, the pyruvic acid content decreased significantly in the G6 h vs. the G0 h group, and in the M6 h vs. the M0 h group. In addition, the concentration of citric acid and fumaric acid was elevated in the M6 h vs. the M0 h, and in the G6.h vs. the G0 h groups. Citric acid and fumaric acid are TCA cycle intermediates. The decline in pyruvic acid and increase in citric acid and fumaric acid indicates the TCA cycle was enhanced, improving ATP production in the gill and mantle tissues of *C. farreri* to overcome heat stress. Increases in citric acid and fumaric acid levels following exposure to high temperatures have also been reported in *S. Schlegelii*, *Carassius carassius* and *Agasicles hygrophila* [31,32,33]. In addition, pyruvic acid is the key substance that links carbohydrate metabolism with amino acid metabolism. Pyruvic acid is a precursor of isoleucine, which is both a ketogenic and a glycogenic amino acid. Isoleucine can enter the TCA cycle to produce ATP through catabolism, which is a more efficient pathway than those of other amino acids, especially under a stress state [34]. In our study, L-isoleucine significantly increased in the mantle tissue, which indicated much higher ATP production in *C. farreri* when under heat stress.

Short-term heat stress also affected lipid metabolism in the mantle tissue of *C. farreri*, mainly involving primary bile acid biosynthesis pathways. The concentrations of glycine and glycocholic acid were significantly enhanced in this study. Glycine can combine with cholic acid to form glycocholic acid, which can promote the digestion and absorption of lipid [35]. Previous research has indicated that lipid provides more energy than the same amount of glycogen or protein, and is the most effective energy source in bivalve shellfish [36]. As a result, we speculate that the enhancement of glycine and glycocholic acid levels may also supply the energy demand for *C. farreri* under heat stress.

Long-term heat stress affected pathways associated with the metabolism of amino acids in mantle and gill tissues. The annotated SDMs contained large amounts of free amino acids (L-arginine, L-histidine, L-tryptophan, L-aspartic acid, L-isoleucine, serine and glycine) and amino acid derivates (taurine). L-arginine, L-histidine, L-tryptophan, L-aspartic acid, L-isoleucine, glycine, serine and taurine concentrations were evidently increased, but those of L-glutamine and β-alanine were not. In *Carassius auratus*, *Haliotis fulgens*, *Eupolyphaga sinensis* the free amino acid levels increase under heat stress [37,38,39]. One study reported that free amino acids could be an energy source during periods of prolonged stress (15 °C low temperature and waterless stress for 5 h) in pearl gentian grouper (♀ *Epinephelus fuscoguttatus* *×* ♂ *Epinephelus lanceolatus*) [40]. Based on the above results, we speculate that the changes in amino acid concentrations would result in the production of more energy to maintain balance between energy supply and demand.

All of the metabolites mentioned above indicate the enhancement of ATP production. However, the ATP concentration in the mantle and gill tissues of *C. farreri* fluctuated, but was not significantly changed in this study. We speculate that although much more energy was demanded, *C. farreri* could maintain energy homeostasis by enhancing carbohydrate, amino acid and lipid metabolism when subjected to 27 °C heat stress, which indicated that *C. farreri* has the potential for adaptation to thermal stress.

### 4.2. Antioxidant Ability

It has been reported that high temperatures can cause the generation of oxygen radicals in marine organisms [41]. For example, overproduction of oxygen radicals occurred in the gills and digestive glands of oysters (*Crassostrea virginica*) when they were exposed to high temperatures (22, 26 and 30 °C) [42]. Elevated levels of oxygen radicals can destroy cellular homeostasis by initiating lipid peroxidation and cause the inhibition of enzymes, which may ultimately result in cell death [43,44,45]. To protect tissues from the effects of oxygen radicals, organisms possess antioxidants (T-AOC, SOD, CAT, GST, etc.) that act as a defense mechanism [46]. In our study, the concentration of T-AOC first increased and then decreased. However, the change in SOD activity was the opposite to that of T-AOC. Moreover, there were no significant differences in the activity of CAT or GST in mantle and gill tissues, compared with the control. These results were in accordance with the reported antioxidant activity of muscle tissue in juvenile hybrid grouper (♀ *E. fuscoguttatus* *×* ♂ *E. lanceolatus*) fed with oxidized oil; these researchers deemed that SOD is reduced in response to oxidative stress, but from an overall point of view, T-AOC is enhanced with an increased level of oxidative stress [47]. Furthermore, the SOD activity showed a certain downward trend in the liver of *Siniperca chuatsi* subjected to temperature stress [6]. Therefore, we consider that the decreased level of SOD activity may be due to the consumption of SOD, which is used to remove oxygen radicals and improve antioxidant capacity.

Lipid peroxidation, caused by excess production of oxygen radicals and MDA in tissues, is an indicator of cellular damage [48,49,50]. In this research, MDA content did not dramatically vary, but it declined in mantle and gill tissues following the extension of heat stress, which was suggestive of lipid peroxidation and cellular damage being relieved. In addition, studies have reported that β-alanine by promoting the formation of pantothenic acid and glutamine could protect the creatures from oxidative stress [51,52,53]. Overall, the results indicated that *C. farreri* can regulate antioxidant activity (T-AOC, SOD, CAT, etc.) and metabolites such as glutamine and β-alanine to avoid oxidative damage.

### 4.3. Metabolic States

In rainbow trout (*Oncorhynchus mykiss*), exposure to high temperatures leads to a reduction in oxygen partial pressure, which triggers a switch to anaerobic metabolism [54]. In *Paralichthys olivaceus* and *Pecten Maximus*, some anaerobic metabolites have similar research reports, such as lactic acid and succinic acid showed significant increase response to thermal stress [3,55]. Unfortunately, in the present research, the anaerobic metabolites succinic acid, acetic acid and propionate were not detected, probably due to the untargeted metabolomic analysis method. However, it has been found that the glucose–opine pathway plays an important role in the anaerobic metabolism of marine invertebrates, and that opine dehydrogenases (OpDHs) promote the smooth running of this pathway. In shellfish, octopine dehydrogenases (ODH) are mainly OpDHs [56]. Under the action of ODH, pyruvic acid and arginine react with NADH to produce octopine and NAD. In our research, the level of octopine was elevated, while arginine and pyruvic acid were decreased, in both mantle and gill tissues of *C. farreri* subjected to heat stress for 6 h. However, when *C. farreri* was exposed to heat stress for 30 days, octopine decreased significantly while arginine increased in the mantle and gill tissues. These results indicated that *C. farreri* entered into anaerobic metabolism when under short-term heat stress, which consumed arginine and produced octopine; but when under long-term thermal stress, octopine was rarely produced through the consumption of arginine, which implied that the level of anaerobic metabolism decreased. Moreover, other researchers have indicated that no accumulation of the anaerobic metabolites succinic acid and acetic acid was detected in the liver tissue of cod (*Lota lota*) or Antarctic tegillarca granosa (*Laternula elliptica*) after long-term temperature stress [57,58].

## 5. Conclusions

We applied untargeted metabolic profiling using LC-MS/MS spectroscopy to investigate metabolic and pathway changes in *C. farreri* under high-temperature stress. The results indicated that high-temperature stress can interfere with the energy metabolism and antioxidant capacity of *C. farreri*. Alteration of energy metabolism was observed in *C. farreri* under thermal stress. An increase in carbohydrate, amino acid and lipid metabolism demonstrated *C. farreri* has the potential to satisfy its higher energy demand when under 27 °C heat stress. In addition, the high T-AOC in mantle and gill tissues suggested *C. farreri* can avoid oxidative damage by regulating its antioxidant capacity after exposure to a thermal environment. Overall, our findings provide new insights into the regulatory mechanisms of *C. farreri* under heat stress.

## Figures and Tables

**Table 1:** KEGG pathway enrichment results of significantly different metabolites (SDMs) of *C. farreri* under heat stress for 6 h and 30 d.

Groups	Level 1	Level 2	Map Title	Metabolites	*p* Value
G_6 h vs. G_0 h	Metabolism	Carbohydrate metabolism	Citrate cycle (TCA cycle), Pentose and glucuronate interconversions	Pyruvic Acid, Fumaric acid, Citric acid, D-Xylulose 5-phosphate, Pyruvic acid, D-Ribulose 5-phosphate	0.031399
		Amino acid metabolism	Butanoate metabolism	L-Glutamic acid, Diacetyl, Fumaric acid, acetoacetate	0.009674
			Alanine, aspartate and glutamate metabolism	L-Glutamic acid, Adenylosuccinic acid, Fumaric acid, Pyruvic Acid	0.013789
			Tyrosine metabolism	L-Tyrosine, Pyruvic acid, 3-Methoxytyramine, Hydroquinone, Fumaric acid, acetoacetate	0.013789
G_30 d vs. G_0 h	Metabolism	Energy metabolism	Sulfur metabolism	Taurine, Serine	0.024072
M_6 h vs. M_0 h	Metabolism	Lipid metabolism	Primary bile acid biosynthesis	Glycine, Glycocholic Acid, Taurochenodeoxycholic acid, Taurocholic Acid, Taurochenodeoxycholate	0.025934
		Carbohydrate metabolism	Pentose and glucuronate interconversions	D-Xylulose 5-phosphate, Pyruvic Acid, D-Ribulose 5-phosphate	0.028313
		Amino acid metabolism	Valine, leucine and isoleucine biosynthesis	Pyruvic Acid, Citraconic acid, L-Isoleucine	0.028313
		Metabolism of other amino acids	Phosphonate and phosphinate metabolism	Ciliatine, Glycine, Pyruvic Acid	0.028313
M_30 d vs. M_0 h	Genetic Information Processing	Translation	Aminoacyl-tRNA biosynthesis	L-Arginine, L-Histidine, L-Glutamine, L-Tryptophan, Serine, L-Isoleucine, Glycine, L-Aspartic acid, β-Alanine	0.018137

## Data Availability

The data presented in this study are available in the article.
